# Changes in Absolute Contents of Compounds Affecting the Taste and Nutritional Properties of the Flesh of Three Plum Species Throughout Development

**DOI:** 10.3390/foods8100486

**Published:** 2019-10-12

**Authors:** Stefano Moscatello, Tommaso Frioni, Francesca Blasi, Simona Proietti, Luna Pollini, Giuseppa Verducci, Adolfo Rosati, Robert P. Walker, Alberto Battistelli, Lina Cossignani, Franco Famiani

**Affiliations:** 1 Istituto di Ricerca sugli Ecosistemi Terrestri, Consiglio Nazionale delle Ricerche, Viale Marconi 2, 05010 Porano (TR), Italy; stefano.moscatello@cnr.it (S.M.); simona.proietti@cnr.it (S.P.); alberto.battistelli@cnr.it (A.B.); 2 Department of Sustainable Crop Production, Università Cattolica del Sacro Cuore, 29122 Piacenza, Italy; tommaso.frioni@unicatt.it; 3 Department of Pharmaceutical Sciences, University of Perugia, via San Costanzo, 06126 Perugia, Italy; francesca.blasi@unipg.it (F.B.); luna.pollini@studenti.unipg.it (L.P.); giuseppa.verducci@unipg.it (G.V.); 4 Consiglio per la Ricerca in Agricoltura e l’analisi Dell’economia Agraria, Centro di Ricerca Olivicoltura, Frutticoltura e Agrumicoltura (CREA–OFA), via Nursina 2, 06049 Spoleto (PG), Italy; adolfo.rosati@crea.gov.it; 5 Department of Agricultural, Food and Environmental Sciences, University of Perugia, via Borgo XX Giugno 74, 06121 Perugia, Italy; rob.walker@talktalk.net

**Keywords:** antioxidant activity, HPLC, malate, plum, polyphenols, sugars

## Abstract

The characteristics of plum fruits of three different species were investigated throughout their development (including over-ripening). The content of primary and secondary metabolites was expressed as amount per gram DW (dry weight) and per fruit in order to obtain information about the balance between their synthesis and dissimilation at different stages of fruit development. In all the plums, during the first stages of development, glucose was the most abundant sugar, whereas sucrose increased during ripening. There was no decrease in malate content per fruit before the commercial harvesting time of any of the plums, whereas a decrease was observed during over-ripening. In general, both the total phenol content and the contents of individual phenols in the flesh expressed on gram DW decreased throughout development, whereas their content per fruit increased, indicating that these decreases were due to a dilution effect arising from the expansion of the flesh. During the development of the flesh, the increase in the contents of the investigated metabolites per fruit shows that there was no net dissimilation of malate up to commercial harvest and of phenols throughout fruit development. Good correlations between the content of phenols to antioxidant activity were found. Shiro flesh, during the last part of fruit development, had lower total carbohydrate and polyphenol contents, lower antioxidant activities, and a higher malate content than the flesh of the other two genotypes.

## 1. Introduction

A number of trees of the genus Prunus are widely cultivated for their fruits, these include apricots, cherries, peaches, and plums. These fruits are known as stone fruits because the inner part of the fruit wall (endocarp) is lignified to form the stone. Botanically, these fruits are classified as drupes and consist of a thin epicarp (skin), a fleshy mesocarp (flesh), and a lignified endocarp (stone) that usually encloses a single seed [1]. The growth pattern of the whole fruits of most plum cultivars is double-sigmoidal, and three stages of growth can be distinguished [2,3]. In the pericarp, the bulk of cell multiplication occurs during stage I, and during this phase the endocarp approaches its final size. During stage II, the rate of increase in the size of the flesh and skin is low and lignification of the endocarp occurs. The large increase in the size of the fruit during stage III arises largely from the expansion of the parenchyma cells of the flesh and skin [4]. Towards the end of stage III, ripening takes place and a number of characteristics of the flesh and skin change. These include a change in colour (arising from an increase in pigment content), softening, and a large increase in soluble sugar content together with a decrease in the concentration of organic acids [1,5].

Sugars are the most abundant soluble components of the flesh of many ripe fruits, including plums, and the ratio of sugars to organic acids largely determines their taste [6,7]. The bulk of the soluble sugar content of the flesh of ripe plums consists of glucose, fructose, sucrose, and sorbitol. This contrasts with peach and apricot flesh which contains little sorbitol, and cherry flesh which contains little sucrose [1,8,9,10,11]. However, there is a considerable variation between plum cultivars in both the total soluble carbohydrate content per g of flesh fresh weight (FW) and also in the relative proportions of glucose, fructose, sucrose, and sorbitol that make up the total content [5,12,13]. The starch content of the flesh of plum and other stone fruits is very low throughout development [12,14]. In ripe plum flesh, malic and quinic acid account for the bulk of the organic acid content, although there is considerable variation in their contents between plum varieties [12,15]. By contrast, peach flesh also accumulates citric acid [16,17]. During the ripening of many fruits, there is often a decrease in the content per gram FW of flesh of these acids, however, this can be a consequence of a dilution effect arising from fruit growth, and the amount of acids per fruit actually increases (i.e., they are synthesised). Nevertheless, in some fruits, this decrease can also arise from their degradation [6]. Hence, in the flesh of many fruits (including many plums), it is not clear whether there is a synthesis and/or degradation of organic acids during ripening. More information on this would be useful for a better understanding of the metabolism of organic acids during ripening.

Compounds that possess antioxidant activity, such as polyphenols, carotenoids, vitamin C, and tocopherols [18,19,20,21,22,23,24,25], are nutritionally important constituents of fruits. Thus, increased amounts of these antioxidants in the human diet have been implicated in decreasing the risk of some cancers and some cardiovascular, atherogenic, and neurological diseases amongst others [26,27,28]. However, very little is known about the contents of these antioxidants in plum flesh and how these contents change during fruit growth and ripening. Only two studies evaluated the changes in contents of these compounds during fruit development, and the results are contradictory. Indeed, one study reported an increase in these compounds throughout fruit development [27], whilst another found a decrease [29]. Therefore, clarification of these differences is required. Furthermore, the decrease in the content per gram FW or dry weight (DW) of these compounds during fruit development could be the result of their degradation, but in the case of the former it could also be the consequence of a dilution effect caused by the growth of the flesh or in the latter case by the accumulation of compounds other than the one which is being studied. More information on these aspects is required in order to establish whether there is degradation or synthesis of these compounds during fruit development.

Previous studies regarding the changes in the contents of both primary and secondary metabolites (carbohydrates, malic acid, and antioxidants) have only investigated in the period up to commercial harvest [1,15,29]. In this study, the contents of these metabolites were determined throughout the entire natural period of fruit growth on the tree (that is, until the drop of the fruits from the tree), and we refer to this previously unstudied extra period as the over–ripening period,

There are a number of species of plums and various hybrids between some of these, however, the most important in terms of the economic value of the crop are the European plum (*Prunus domestica*) and the Japanese plum (*Prunus salicina*) [30]. Thus, this diverse genetic composition is likely to affect the content of compounds (e.g., soluble carbohydrates, organic acids, and antioxidants) present in the flesh of different cultivars.

The aim of the present work was to characterise the changes in contents of sugars and antioxidants in the flesh of plums throughout their whole development (including over-ripening). This was done by determining both the concentration of the considered compounds (on a gram DW basis) and their absolute contents (on a per fruit basis) in order to establish whether there was a degradation or synthesis of these compounds at different stages of development. In order to establish if there was an effect of genotype, three different species/cultivars were studied: a European plum cultivar, a Japanese plum cultivar, and a Mirabolano plum (*Prunus cerasifera*). To minimise the effects of soil/climatic conditions and cultivation practices on the contents of the studied compounds, all fruits were sampled from trees growing in the same orchard and during the same season.

## 2. Materials and Methods

### 2.1. Chemicals

The analytical standards 2,2′-azino-bis(3-ethylbenzothiazoline-6-sulphonic acid) diammonium salt (ABTS), 2,2-diphenyl-1-picrylhydrazyl (DPPH radical), Folin and Ciocalteu’s phenol reagent, gallic acid, (±)-6-hydroxy-2,5,7,8-tetramethylchromane-2-carboxylic acid (Trolox), 2,4,6-tripyridyl-s-triazine, and sorbitol were from Sigma–Aldrich (Milan, Italy). (+)-Catechin, chlorogenic acid, kaempferol, kaempferol-3-*O*-glucopyranoside, quercetin, and quercetin-3-*O*-glucopyranoside were obtained from Extrasythese (Genay, France). All the reagents used were of analytical grade. Solvents used for analyses were HPLC grade and purchased from VWR (Milan, Italy). Deionized water was used throughout and obtained using a Milli-Q™ system (Millipore Corp, Billerica, MA, America).

### 2.2. Plant Material

In 2013, fruits of three different plum species, namely, *Prunus cerasifera* (Mirabolano), *Prunus domestica* cultivar President, and *Prunus salicina* cultivar Shiro, were collected every 1–2 weeks from fruit-set to the end of their development. Specifically, from 20 to 155 days after full bloom (AFB) for Mirabolano, from 25 to 180 AFB for President, and from 25 to 150 AFB for Shiro. Plums were collected from plants growing in the experimental orchard of the Department of Agricultural, Food, and Environmental Sciences of the University of Perugia, in Deruta (Perugia), central Italy. Antiradical activities, measured as Trolox equivalent antioxidant capacity (TEAC) and DPPH free radical-scavenging assay and HPLC analyses, were determined at five stages of development, these are shown in Table 1. For all sampling times, only healthy fruits were used and these were taken from several positions on the plant.

### 2.3. Measurement of Fresh and Dry Weights

For each species and at each sampling time, the individual weights of 15 intact plums, together with the weights for each of these plums of their mesocarp plus epicarp (flesh), were determined. This was done for both freshly harvested material and for material dried to constant weight in a drying oven (Binder FD 53, Tuttlingen, Germany) at 90 °C.

### 2.4. Measurement of Flesh Firmness and Soluble Solids Content

For each species and starting at the beginning of stage III of fruit development, the flesh firmness of 15 plums was determined at each time point. This was done after the removal of about 1 cm^2^ of skin, using a hand-held penetrometer (Effe.gi, Ravenna, Italy) equipped with an 8-mm plunger. The soluble solids contents of the flesh of the same fruits (expressed as °Brix) was also measured. This was done by taking a sample of juice from the equatorial part of the flesh of each fruit, the °Brix was then measured using a hand-held refractometer (Model M, Atago, Fukaya, Japan).

### 2.5. Preparation of a Lyophilized Powder

For the measurements of sugar, malate, and phenolics, a lyophilized powder was prepared to ensure that samples were representative of a number of fruits. For each species and stage of development, three samples, each represented by portion of flesh from 10–20 fruits, were mixed and then freeze-dried with a lyophilizator (LIO5PDGT, Cinquepascal, Milano, Italy). Then, the freeze-dried material was ground to a powder (using a lab mill) which was used for the subsequent analyses.

### 2.6. Measurement of Sugar and Malate Content

Glucose, fructose, sucrose, and starch were measured using an enzyme-coupled spectrophotometric method [31]. Sorbitol was determined as previously reported [32]. Malate was measured using an enzyme-coupled method [1].

### 2.7. Determination of Total Phenol Content (TPC)

Phenolic compounds were extracted from powders employing pure methanol in an ultrasonic bath [33]. The TPC was determined spectrophotometrically according to the method reported in a previous paper [34]. This procedure uses Folin and Ciocalteu’s phenol reagent, and the absorbance is determined at 765 nm. The TPC was expressed as mg of gallic acid equivalents (GAE) per gram of dry weight (mg GAE g^−1^ DW) and as mg GAE fruit^−1^.

### 2.8. Determination of In Vitro Antioxidant Activity

#### 2.8.1. Ferric Reducing Antioxidant Power (Frap) Assay

The spectrophotometric FRAP assay was carried out as previously reported [35], and absorbance at 600 nm was measured. The antioxidant activity of plum flesh was expressed as Trolox equivalents (TE) per gram of dry weight (µmol TE g^−1^ DW) and as mg TE fruit^−1^.

#### 2.8.2. Trolox Equivalent Antioxidant Capacity (Teac) Assay-ABTS

The TEAC assay was carried out as reported in a previous paper [36], applying some slight modifications [37]. The antiradical activity against ABTS radical cation (ABTS^•+^) was obtained, measuring the absorbance at 734 nm. The antioxidant capacity of each sample was expressed as Trolox equivalents (TE) per gram of dry weight (mg TE g^−1^ DW) and as mg TE fruit^−1^.

#### 2.8.3. DPPH Free Radical-Scavenging Assay

The DPPH free radical-scavenging activity was determined according to a spectrophotometric procedure, measuring the absorbance at 517 nm [38]. The percentage of antioxidant activity (AA%) for each sample was calculated using the following formula: AA% = (Absc − Abss/Absc) × 100 (Absc = absorbance of the control solution containing only DPPH radical; Abss = absorbance of the DPPH solution containing sample). IC50, the sample concentration that gave 50% inhibition of DPPH radical, was calculated using the regression equation obtained by plotting AA% against sample concentration.

### 2.9. HPLC Analysis of Phenol Compounds

The HPLC analyses were performed using a Thermo Spectra Series pump (Thermo Scientific, Rockford, IL, USA), a Hypersil GOLD column (3 μm particle size, 150 × 4.6 mm i.d., Thermo Scientific), and a Spectra System UV6000LP Diode Array Detector (Thermo Scientific), as reported previously [39]. The solvents were (A) 0.1% formic acid in water and (B) 0.1% formic acid in acetonitrile. The samples were analyzed by gradient elution at a flow rate of 1.0 mL min^−1^. The initial mobile phase composition was 95% of A. The percentage of B was linearly increased to 20% at 30 min and to 55% at 50 min. The injection volume was 20 µL. Detection was performed on-line by measuring absorbance at a range of wavelengths between 190–390 nm. The chromatograms were acquired and the data handled using Xcalibur software version 1.2 (Finnigan Corporation 1998–2000, San Jose, CA, USA). Standard solutions were used to identify and quantify the eluted material (an example profile is shown in Figure S1). For each standard, calibration curves were obtained by applying it to the column at different concentrations ranging from 1.5 µg mL^−1^ to 120.0 µg mL^−1^. The validation parameters are reported in Table S1. The identification of phenol compounds was performed by HPLC-tandem mass spectrometry, as reported in a previous paper [40].

### 2.10. Statistical Analysis

All measurements were carried out in triplicate and the results were expressed as mean value and standard error. Correlation analyses have been performed using Origin 5.0 (Microcal™ Software Inc., Northampton, MA, USA).

## 3. Results and Discussion

### 3.1. Physical and Chemical Characteristics of the Flesh during Development

**Figure 1:**
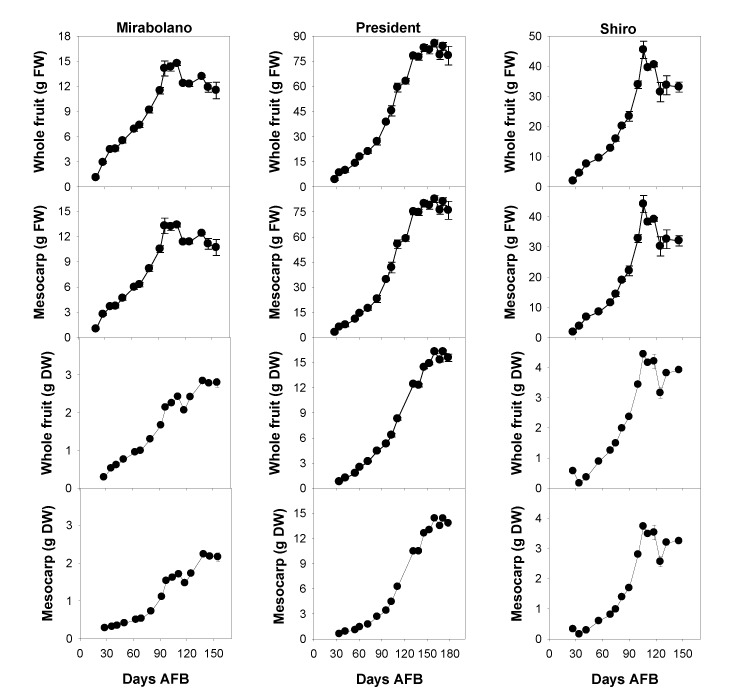
Fresh and dry weights of both the whole fruit and its flesh (mesocarp + epicarp) of *Prunus cerasifera*—Mirabolano, *Prunus domestica*—cultivar President, and *Prunus salicina*—cultivar Shiro at different stages of development. Each point on the graph shows the mean and standard error, *n* = 15. AFB = after full bloom.

The changes in the fresh weights (FW) and dry weights (DW) of both whole fruits and the flesh of each plum species is shown in Figure 1. Both FW and DW continuously increased up to 95–105 days AFB (Mirabolano), 155 days AFB (President), and 100–110 days AFB (Shiro). Subsequently, the FW either remained constant (President) or decreased (Mirabolano and Shiro). This decrease in FW was likely due to the heaviest over-ripened fruits dropping from the tree.

**Figure 2:**
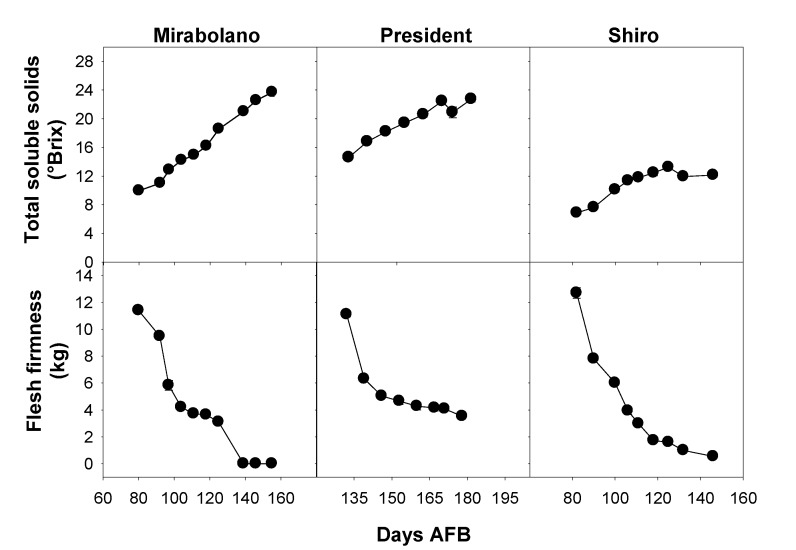
Soluble solids content and flesh (mesocarp) firmness of the flesh (mesocarp + epicarp) of fruits of *Prunus cerasifera*—Mirabolano, *Prunus domestica*—cultivar President, and *Prunus salicina*—cultivar Shiro at different stages of development. Each point on the graph shows the mean and standard error, *n* = 15. AFB = after full bloom.

The flesh firmness and total soluble solids (TSS) content of fruits are both harvesting indexes that are used commercially to determine when to pick the crop (commercial harvesting) [41]. Further, they give a useful indication of the crop quality [42]. Throughout the development of the fruits of each of the plum species, flesh firmness decreased whereas TSS increased (Figure 2). The flesh of the fruits of both Mirabolano and Shiro was very soft in the final part of development, and flesh firmness was very close to zero. The maximum TSS content of the flesh of fruits of President and Mirabolano (23 and 24 °Brix, respectively) was much higher than that of Shiro (12 °Brix) (Figure 2). Shiro plums are usually harvested when the TSS is in the range 10–11 °Brix and the flesh firmness is around 3 N; while President plums are harvested when the TSS is 18–19 °Brix and the flesh firmness is 4–5 N [41]. This is because the consistency of the flesh is suitable for the transport, storage, and subsequent sale of the fruits, and the TSS is high enough to impart on the fruits a good taste. In the present study, fruits were at the stage of development that is the stage of commercial harvesting, at around 110 days AFB (Shiro) and 155 days AFB (President). These times corresponded to the first half of July (Shiro) and the end of the first half of September (President). Mirabolano is normally harvested during the first half of July, and this time corresponded to 95–105 days AFB.

It is interesting to note that the time of commercial harvest corresponds to the end of fruit growth in terms of the increase in both fresh and dry weights (Figure 1 and Figure 2).

### 3.2. Carbohydrate Content of the Flesh during Development

**Figure 3:**
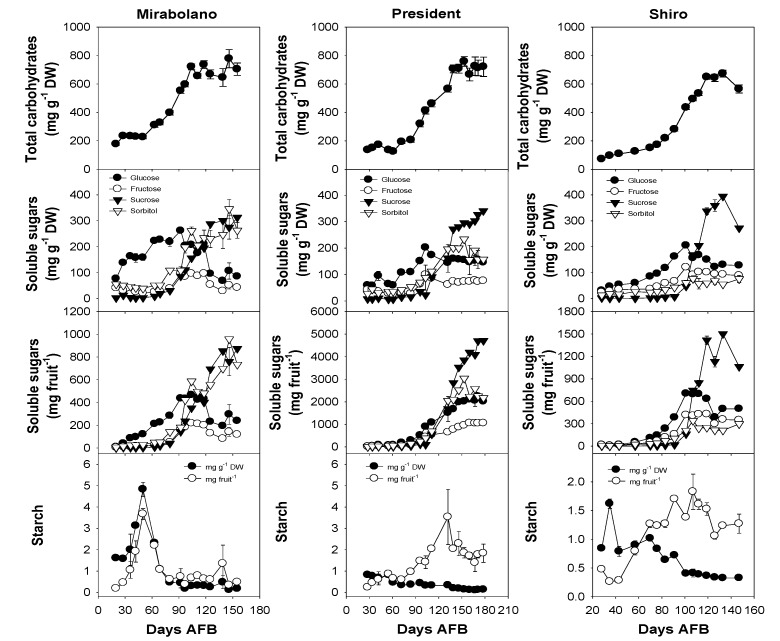
Soluble carbohydrate contents of the flesh (mesocarp + epicarp) of fruits of *Prunus cerasifera*—Mirabolano, *Prunus domestica*—cultivar President, and *Prunus salicina*—cultivar Shiro at different stages of development. Each point on the graph shows the mean and standard error, *n* = 3. AFB = after full bloom.

Figure 3 shows the changes in the contents of soluble carbohydrates and starch in the flesh of Mirabolano President and Shiro during development. In the flesh of all the plums during early stages of development, the most abundant soluble sugar was glucose, and Mirabolano flesh had the highest content. Glucose content increased up to about 90 days AFB in Mirabolano, 100 days in Shiro, and 105 days AFB in President, and then decreased in all them. Fructose content was lower than that of glucose in the flesh of all the plums. The content of fructose increased up to 100–105 days AFB, and then decreased to a constant amount (President and Shiro) or continuously decreased (Mirabolano). The glucose content of both damson plum flesh (*Prunus domestica*) and certain Japanese plum cultivars is higher than that of fructose [15,29,43]. However, in some cultivars of Japanese plum, the fructose content of the flesh is higher than that of glucose throughout development [12]. The sucrose content was very low in the young flesh of all plums, however, the content increased greatly between 65–125 days AFB (Mirabolano), 85–140 days AFB (President), and 90–120 days AFB (Shiro). After these times, sucrose content increased at a lower rate in the flesh of each plum. In the young flesh of Mirabolano, the second most abundant soluble carbohydrate was sorbitol, whose content then increased to an amount that was similar to that of sucrose. A different trend in sorbitol content was observed in Shiro flesh, in which this content only changed slightly during development. The maximum sorbitol content of the ripe flesh differed greatly between the plum species and was much higher in Mirabolano.

In the young flesh of all the plums, a low amount of starch (less than 5 mg g^−1^ DW) was present. The contents of glucose, fructose, sorbitol, and sucrose expressed as mg fruit^−1^ showed patterns similar to those expressed as mg g^−1^ DW.

### 3.3. Malate Content of the Flesh during Development

**Figure 4:**
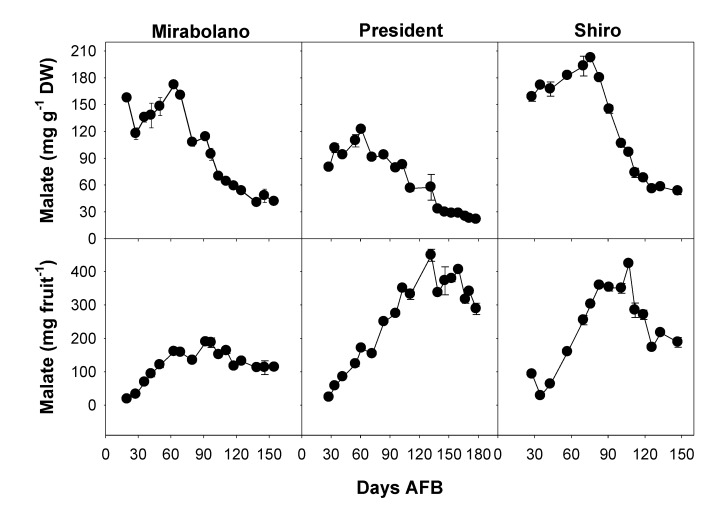
Malate contents of the flesh (mesocarp + epicarp) of fruits of *Prunus cerasifera*—Mirabolano, *Prunus domestica*—cultivar President, and *Prunus salicina*—cultivar Shiro at different stages of development. Each point on the graph shows the mean and standard error, *n* = 3. AFB = after full bloom.

Malic together with quinic acid accounts for the bulk of the organic acid content of ripe plum flesh [1,6,7]. Malate content (mg g^−1^ DW) increased up to about 65 days AFB (Mirabolano and President) and 75 days AFB (Shiro), and after these times, decreased (Figure 4). Shiro flesh possessed the highest content and President flesh the lowest. Malate content (mg fruit^−1^) increased up to about 90 days AFB (Mirabolano), 130 days AFB (President), and about 110 days AFB (Shiro), and then decreased in each plum. These abundances and changes in content during development are comparable to those reported for Damson plum and Japanese Amber Jewel plum [12,15].

In the present study, the observation that malate content per fruit did not decrease up to commercial harvest shows that there was no net dissimilation of this compound, and that the decrease in its concentration (mg g^−1^ DW) was a dilution effect arising from the large increase in fruit weight [8,44,45]. A net dissimilation of malate only occurred after the time of commercial harvesting, that is during over-ripening.

### 3.4. Determination of Total Phenol Content

**Figure 5:**
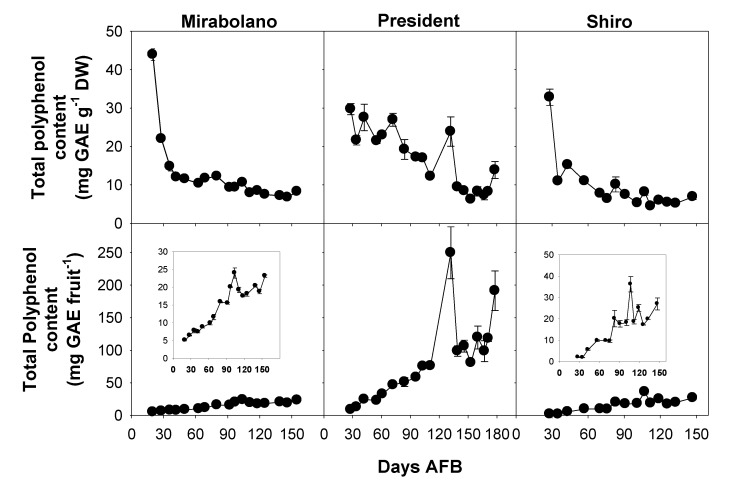
Total polyphenol contents of the flesh (mesocarp + epicarp) of fruits of *Prunus cerasifera*—Mirabolano, *Prunus domestica*—cultivar President, and *Prunus salicina*—cultivar Shiro at different stages of development. Each point on the graph shows the mean and standard error, *n* = 3. AFB = after full bloom.

In all plums the TPC content per g DW was high during the early part of development and then decreased (Figure 5). This is in agreement with the results reported by some authors [29], but contrasts with others [27], who found that the TPC per FW increased during the final weeks of development.

The TPC, expressed as mg GAE per fruit, increased during development, and the contents had a peak at about 100 days AFB (Mirabolano), 130 days AFB (President), and 110 days AFB (Shiro) (Figure 5). For each plum, these times were around the date of commercial harvesting. The TPC of ripe plum flesh can be quite variable, and a content of 61.9 mg GAE 100 g^−1^ FW was reported for Normal plums and 209 mg GAE 100 g^−1^ FW for Kuhpanz plum [46]. The increase of the TPC (mg GAE fruit^−1^) during ripening/over-ripening shows that these compounds were synthesised throughout development (Figure 5). Thus, the decrease in the TPC per gram DW during later stages of ripening was a dilution effect brought about by the large increase in the DW, and the latter largely arose from the accumulation of soluble carbohydrates (Figure 3).

### 3.5. Evaluation of Antioxidant Activity

**Figure 6:**
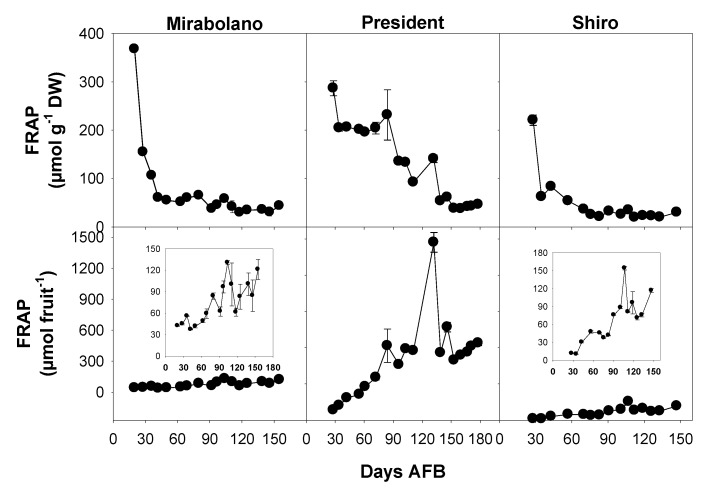
Antioxidant activities of the flesh (mesocarp + epicarp), expressed as ferric reducing antioxidant power (FRAP), of fruits of *Prunus cerasifera*—Mirabolano, *Prunus domestica*—cultivar President, and *Prunus salicina*—cultivar Shiro at different stages of development. Each point on the graph shows the mean and standard error, *n* = 3. AFB = after full bloom.

The antioxidant capacity of plum flesh was measured using three different methods. These were the ABTS and DPPH assays which determined antiradical activity, and the FRAP assay which determined reducing power. Phenolic compounds can act as antioxidants and their inclusion in the human diet can potentially both reduce the formation of free radicals and also remove them from various cell types in humans. Free radicals have deleterious effects on human health and reducing their content in vivo might contribute to reducing the risks of certain diseases [28]. For each plum species, the reducing capacity measured by FRAP and the antiradical activity measured by ABTS assays are reported in Figure 6 and Table 2, respectively.

The results expressed per gram were highest early in development and then progressively decreased, on the contrary, an increasing trend was observed when the data were expressed on per fruit. Moreover, when the antiradical activity was measured by DPPH assay and expressed as IC50 (Table 2), an increase was observed during the development.

It is important to point out that this trend corresponds to a decrease of antiradical activity, as IC50 represents the sample concentration that gives 50% inhibition of DPPH radical. The results obtained for antioxidant activity during plum development are in agreement with a recent paper [47], showing a decreasing trend of antiradical activity, measured as peroxyl radical scavenging capacity, and expressed as µmol ascorbic acid equivalents 100 g^−1^ FW during Sanhua plum (*P. salicina*) development. Other authors studied antioxidant activity of Myrobalan plum using FRAP assay and reported values ranging from 11.20 to 44.83 μmol TE g^−1^ FW [48].

### 3.6. HPLC-DAD Analysis of Phenol Compounds

Phenol composition of the flesh of the three plum species at different stages of development is shown in Table 3. As an example, the HPLC-DAD profiles of the M3, P3, and S3 samples are reported in Figures S2–S4, respectively. The chlorogenic acids were the most abundant group of phenols, and neochlorogenic acid (a member of this group) was the most abundant of these in the flesh of each plum during most of their development. This is in agreement with the results obtained for seventeen plum cultivars [46] and for several Japanese plum cultivars [49]. During development, the content of neochlorogenic acid in the flesh decreased from 5432.75–980.55 µg g^−1^ DW (Mirabolano), 9643.08–1972.11 µg g^−1^ DW (President), and 1452.55–702.73 µg g^−1^ DW (Shiro). In general, there was also a decrease in the content of chlorogenic acid in the flesh of each plum during development, however, in President and Shiro flesh, the content increased slightly towards the end of their growth. Similarly, some authors reported a decreasing content (mg 100 g^−1^ FW) for other phenolic compounds—among which was epicatechin—in the flesh of the Japanese Sanhua plum during development [47]. By contrast, other authors found no decrease in the phenolic content (including the contents of neochlorogenic and chlorogenic acid, expressed as mg 100 g^−1^ FW) of four European plum cultivars during a period ranging from 25 to 33 days [33]. Catechin content (µg g^−1^ DW) was greatest in the flesh of each plum early in development (Table 3). Catechin has previously been detected in the flesh of several plum cultivars [46,49], while epicatechin has been detected in the flesh of both Chinese plum (*P. salicina*) [50] and Sanhua plum [47].

Glycosylated kaempferol was only detected in Mirabolano and Shiro plums, glycosylated quercetin was present in all, whereas the unglycosylated forms were only detected in Shiro (Table 3). In a previous paper, glycosylated quercetin was found in all the investigated plum cultivars, whereas glycosylated kaempferol was found in only some [46]. The unglycosylated forms of quercetin and kaempferol have been detected in Black Amber plums [51] and Chinese plum cultivars [50]. For all the phenolic compounds, the content per fruit (Table 4) did not decrease during development, and the pattern was the opposite to that of content per gram DW. This difference was due to a dilution effect arising from the accumulation of large amounts of sugars during the development of the flesh (Figure 3). Importantly this shows that there was a net synthesis and not catabolism of these compounds during the development and ripening of the flesh of each plum, as it could be believed from the only observation of the content per gram DW.

### 3.7. Phenolic Compounds and Antioxidant Activity: Correlations

Table 5 gives the determination coefficients (*R*^2^) obtained from correlations between the results from spectrophotometric (TPC and the antioxidant in vitro procedures: ABTS, DPPH, and FRAP) and HPLC analyses (TPH, total phenol and NA, neochlorogenic acid, measured by HPLC analysis).

In general, good correlations between parameters measured using spectrophotometric procedures was observed (Table 5). High correlations were found between TPC and FRAP (*p* ≤ 0.001 for Mirabolano, *p* ≤ 0.01 for President and Shiro), while TPC was correlated with ABTS and DPPH especially in President (*p* ≤ 0.01). Good correlations were found between ABTS and FRAP (*p* ≤ 0.01 for Mirabolano, *p* ≤ 0.001 for President). Strong relationships between TPC and antioxidant capacity were also found for other fruit species, such as sweet cherry [52], blackberry, and hybrid berry cultivars [53]. The correlation between the contents of phenolic compounds determined by HPLC and in vitro assays was evaluated, and generally strong correlations were noted, in particular for ABTS (*p* ≤ 0.01 for the three species) (Table 5).

Additionally, for neochlorogenic acid, good correlations were found when considering ABTS and FRAP (*p* ≤ 0.01 for Mirabolano and President). Similarly, for plums grown in California, some authors found good correlations between total phenolic contents measured by HPLC and results obtained using both the DPPH and FRAP assays [54]. The results of the present study indicate that phenolic compounds make a major contribution to the total antioxidant activities of the flesh of all three considered plum species.

### 3.8. Changes during Development, Including Over-Ripening

A consideration of sugar and malate contents (Figure 3 and Figure 4) indicated that ripening started around 65–70 days AFB (Mirabolano), 65–85 days AFB (President), and 75–90 days AFB (Shiro). Thus, at these times, malate contents per gram DW had reached their maximum values and started to decrease; and soluble sugar contents started to increase greatly. The harvesting indexes (based on either flesh firmness or total soluble solids) indicated that the time of commercial harvesting was 95–105 days AFB (Mirabolano), around 155 days AFB (President), and around 110 days AFB (Shiro) (Figure 2). Fruits were left on the trees after the time of commercial harvesting (after these times, the period defined in this paper as over-ripening started). During the over-ripening period, flesh firmness decreased to very low values especially in Mirabolano and Shiro, and in all three cultivars TSS increased to about 12 °Brix (Shiro), 23 °Brix (President), and 24 °Brix (Mirabolano). Soluble sugar contents and especially that of sucrose also tended to increase. Further, during over-ripening, there were differences in sorbitol content between cultivars, and its content was similar to that of sucrose in Mirabolano, was the second most abundant sugar in President, and was relatively low in Shiro. In each cultivar, malate content per fruit decreased during over-ripening, and this showed that it was metabolised. In general, TPC, antioxidant activities, and contents of individual phenolics per gram DW decreased during development; whereas the activities and contents per fruit increased. There were only very few exceptions to these patterns of changes, at the last development stage, the contents per gram DW of catechin and both glycosylated and free quercetin and kaempferol (Table 3) were higher than at the previous developmental stage.

## 4. Conclusions

The changes in contents of soluble sugars, organic acids, and antioxidant compounds (including the activities of the latter) in the flesh of three species of plum (*P. cerasifera*—Mirabolano, *P. domestica* cultivar President, and *P. salicina* cultivar Shiro) were determined throughout development, including the over-ripening period. As far as we are aware, this is the first study in which the whole fruit development has been considered. Many previous studies of the contents of these metabolites in plum flesh have only used ripe fruits. In addition, we expressed metabolite contents as both amount per gram DW and absolute content per fruit. This is important because when content is expressed on a per gram DW basis this can give the false impression that there is a degradation of a metabolite, however, by comparing with the changes in amount per whole flesh (absolute content per fruit), whether the decrease is a result of a dilution effect arising from the accumulation of large amounts of sugars is revealed. As far as we are aware, this is the first study on plum flesh in which this comparison has been done for secondary metabolites and antioxidant activities. Further, this comparison has not been done for sugars and malic acid in the over-ripening period. This approach has allowed us to establish that malic acid was synthesised up to commercial harvest and that a net dissimilation of malic acid occurred only during the over-ripening period. Moreover, this comparison showed that the decrease in contents per gram DW of phenols during fruit development was generally due to a dilution effect, because the amounts per fruit increased throughout fruit development and ripening. This means that there was no dissimilation but synthesis of these metabolites during the whole fruit growth and ripening period. Such information is of fundamental importance when considering strategies that may be of use in increasing the contents of these metabolites in ripe plum flesh or when studies on the metabolism of these compounds are performed. The use of three different species/cultivars has also allowed us to show some differences in the variation patterns of some soluble sugars and phenolic compounds due to the genotype.

The present study shows that determinations of changes in the contents of compounds in the flesh of fruits during development must take into account dilution effects arising from growth. To do this, contents should be expressed on both a whole fruit and a per gram of FW or DW basis.

## Figures and Tables

**Table 1:** Stages of development of the plum samples.

	**Mirabolano**				
	M1	M2	M3	M4	M5
Days AFB *	28	63	80	111	146
Days AFB	**President**				
	P1	P2	P3	P4	P5
	34	72	111	153	178
Days AFB	**Shiro**				
	S1	S2	S3	S4	S5
	35	57	91	107	133

* AFB: after full bloom

**Table 2:** Antiradical activities (mean value ± standard error, *n* = 3) of the fruit flesh of the three plum species at different development stages.

Plum Species	Samples *	ABTS	ABTS	DPPH
		mg TE g^−1^	mg TE fruit^−1^	IC50
Mirabolano	M1	18.10 ± 0.07	5.25 ± 0.02	7.49 ± 0.02
	M2	7.03 ± 0.16	6.69 ± 0.16	22.78 ± 0.38
	M3	6.04 ± 0.52	7.82 ± 0.68	31.11 ± 1.31
	M4	4.69 ± 0.08	11.36 ± 0.18	76.46 ± 2.52
	M5	6.44 ± 0.12	17.86 ± 0.34	56.22 ± 0.61
President	P1	15.16 ± 0.88	2.45 ± 0.14	8.54 ± 0.39
	P2	15.53 ± 0.26	13.69 ± 0.23	4.22 ± 0.16
	P3	7.27 ± 0.14	17.15 ± 0.33	15.56 ± 1.02
	P4	5.54 ± 0.99	24.57 ± 4.38	30.35 ± 1.71
	P5	4.92 ± 0.61	18.76 ± 2.31	28.63 ± 2.40
Shiro	S1	10.40 ± 0.04	6.16 ± 0.03	3.35 ± 0.71
	S2	7.96 ± 0.14	13.79 ± 0.24	12.48 ± 0.76
	S3	3.61 ± 0.07	22.51 ± 0.44	42.28 ± 1.41
	S4	3.57 ± 0.01	46.48 ± 0.11	75.50 ± 0.12
	S5	4.55 ± 0.46	62.77 ± 6.35	56.51 ± 4.41

* Plum sample abbreviations are reported in Table 1. ABTS: 2,2′-azino-bis(3-ethylbenzothiazoline-6-sulphonic acid) diammonium salt. DPPH: 2,2-diphenyl-1-picrylhydrazyl.

**Table 3:** Content of phenolic compounds (μg g^−1^ DW; mean value ± standard error, *n* = 3) in the fruit flesh of the three species of plum* at different development stages.

μg g^−1^	Neochlorogenic Acid	Catechin	Chlorogenic Acid	Chlorogenic Acid Derivatives ^§^	Quercetin-3-*O*-Glucosides	Kaempferol-3-*O*-Glucosides	Quercetin	Kaempferol
**M1**	5432.75 ± 92.53	2645.76 ± 53.30	2250.68 ± 25.73	2076.06 ± 1.03	nd	45.99 ± 1.30	nd	nd
**M2**	2188.59 ± 29.66	444.40 ± 16.77	1354.11 ± 5.45	903.20 ± 1.33	nd	40.99 ± 0.75	nd	nd
**M3**	1490.47 ± 3.50	215.18 ± 8.17	949.87 ± 8.19	664.35 ± 16.41	23.11 ± 0.44	33.80 ± 0.66	nd	nd
**M4**	1093.74 ± 3.28	161.37 ± 2.71	832.94 ± 3.84	605.16 ± 14.69	143.51 ± 1.83	25.38 ± 0.70	nd	nd
**M5**	980.55 ± 1.04	67.01 ± 1.03	784.78 ± 4.19	370.46 ± 2.24	184.00 ± 2.82	27.22 ± 0.30	nd	nd
**P1**	9643.08 ± 25.77	2873.01 ± 16.90	4319.22 ± 53.55	2955.25 ± 30.51	206.04 ± 2.16	nd	nd	nd
**P2**	7751.09 ± 99.35	2471.09 ± 14.53	2151.47 ± 13.74	2558.12 ± 2.35	46.89 ± 0.20	nd	nd	nd
**P3**	3129.33 ± 12.37	969.02 ± 15.45	1323.68 ± 9.68	1236.08 ± 20.53	45.80 ± 0.67	nd	nd	nd
**P4**	1993.27 ± 21.94	469.56 ± 4.03	795.62 ± 1.91	763.47 ± 18.69	41.19 ± 0.39	nd	nd	nd
**P5**	1972.11 ± 2.81	439.70 ± 1.41	826.88 ± 5.11	615.91 ± 4.51	74.28 ± 0.41	nd	nd	nd
**S1**	1452.55 ± 9.93	987.81 ± 12.32	1037.22 ± 7.84	523.46 ± 5.13	471.65 ± 13.70	285.07 ± 7.19	101.40 ± 0.20	306.81 ± 20.21
**S2**	1281.14 ± 1.76	454.64 ± 8.89	1017.91 ± 1.44	563.34 ± 1.51	350.11 ± 30.11	186.64 ± 9.95	91.82 ± 0.51	224.38 ± 1.09
**S3**	773.95 ± 0.80	398.96 ± 0.59	736.46 ± 3.46	197.97 ± 0.29	133.17 ± 0.97	51.45 ± 5.21	nd	126.39 ± 2.15
**S4**	704.74 ± 0.41	128.86 ± 1.38	684.88 ± 3.36	162.41 ± 0.26	51.73 ± 0.29	9.02 ± 0.06	nd	56.79 ± 0.58
**S5**	702.73 ± 2.23	439.55 ± 4.29	728.38 ± 1.92	146.99 ± 1.67	141.06 ± 9.33	65.63 ± 8.28	10.12 ± 1.01	47.25 ± 0.11

* Plum sample abbreviations are reported in Table 1; §: sum of chryptochlorogenic acid, isochlorogenic A, and isochlorogenic C; nd = not detectable.

**Table 4:** Content of phenolic compounds (μg fruit^−1^; mean value ± standard error, *n* = 3) in the fruit flesh of the three species of plum* at different development stages.

μg fruit^−1^	Neochlorogenic Acid	Catechin	Chlorogenic Acid	Chlorogenic Acid Derivatives ^§^	Quercetin-3-*O*-Glucosides	Kaempferol-3-*O*-Glucosides	Quercetin	Kaempferol
**M1**	1576.04 ± 32.97	767.54 ± 18.99	652.92 ± 9.17	602.26 ± 0.37	nd	13.34 ± 0.46	nd	nd
**M2**	2082.00 ± 34.66	422.76 ± 19.60	1288.17 ± 6.37	262.02 ± 0.47	nd	38.99 ± 0.88	nd	nd
**M3**	1930.46 ± 5.58	278.70 ± 13.01	1230.27 ± 13.03	192.73 ± 5.85	29.93 ± 0.69	43.78 ± 1.05	nd	nd
**M4**	2649.69 ± 9.75	390.95 ± 8.06	2017.87 ± 11.43	175.56 ± 5.23	347.67 ± 5.45	61.48 ± 2.10	nd	nd
**M5**	2720.54 ± 3.55	185.92 ± 3.51	2188.98 ± 14.27	107.47 ± 0.80	510.51 ± 9.61	75.53 ± 1.01	nd	nd
**P1**	5716.42 ± 18.76	1703.12 ± 12.31	2560.43 ± 38.99	1751.87 ± 22.22	122.14 ± 1.57	nd	nd	nd
**P2**	13424.89 ± 211.38	4279.93 ± 30.91	3726.35 ± 29.24	1516.45 ± 1.71	81.21 ± 0.43	nd	nd	nd
**P3**	19500.09 ± 94.72	6038.34 ± 118.27	8248.39 ± 74.07	732.75 ± 14.95	285.42 ± 5.12	nd	nd	nd
**P4**	25917.90 ± 350.43	6105.60 ± 64.32	10345.19 ± 30.50	452.59 ± 13.61	535.55 ± 6.26	nd	nd	nd
**P5**	27236.76 ± 47.66	6072.68 ± 23.90	11400.52 ± 86.64	365.11 ± 3.29	1025.85 ± 6.87	nd	nd	nd
**S1**	234.73 ± 1.97	159.63 ± 2.45	167.62 ± 1.56	84.59 ± 1.02	76.22 ± 2.72	46.07 ± 1.43	16.39 ± 0.03	49.58 ± 4.01
**S2**	1129.58 ± 1.90	400.86 ± 9.63	897.49 ± 1.56	91.04 ± 0.30	308.69 ± 32.61	164.56 ± 10.77	80.96 ± 0.46	197.84 ± 1.18
**S3**	1826.98 ± 2.33	941.79 ± 1.73	1738.50 ± 10.03	31.99 ± 0.06	314.35 ± 2.80	121.44 ± 15.10	nd	298.35 ± 6.24
**S4**	3127.20 ± 2.22	571.82 ± 7.51	3039.10 ± 18.31	26.25 ± 0.05	229.56 ± 1.61	40.00 ± 0.33	nd	252.00 ± 3.15
**S5**	2677.35 ± 10.41	1674.63 ± 20.08	2775.05 ± 8.96	23.75 ± 0.33	2442.39 ± 43.65	1012.02 ± 38.74	419.53 ± 3.86	180.01 ± 0.53

* Plum samples abbreviations are described in Table 1; ^§^ sum of chryptochlorogenic acid, isochlorogenic A, and isochlorogenic C; nd = not detectable.

**Table 5:** Results of the correlations (*R*^2^) between phenol content and antioxidant activity *.

**Plum Species**	**TPC/ABTS**	**TPC/DPPH**	**TPC/FRAP**	**ABTS/DPPH**	**ABTS/FRAP**	**DPPH/FRAP**
**Mirabolano**	0.88	0.91	0.99	0.81	0.92	0.66
**President**	0.95	0.99	0.96	0.94	0.99	0.98
**Shiro**	0.63	0.82	0.95	0.82	0.79	0.80
**Plum Species**	**TPH/ABTS**	**TPH/DPPH**	**TPH/FRAP**	**NA/ABTS**	**NA/DPPH**	**NA/FRAP**
**Mirabolano**	0.98	0.95	0.97	0.96	0.95	0.95
**President**	0.96	0.76	0.96	0.95	0.80	0.95
**Shiro**	0.99	0.98	0.91	0.95	1.00	0.91

* The correlations have been calculated using the results expressed per gram DW; TPC, total phenol content determined by spectrophotometric assay; TPH, total phenol content determined by HPLC analysis; NA, neochlorogenic acid.

## Supplementary Materials

The following are available online at https://www.mdpi.com/2304-8158/8/10/486/s1, Figure S1: HPLC-DAD profile of authentic standard mixture (1. Catechin, 2. Chlorogenic acid, 3. Quercetin-3-*O*-glucoside, 4. Kaempferol-3-*O*-glucoside, 5. Quercetin, 6. Kaempferol). Figure S2: HPLC-DAD profile of Mirabolano sample—development stage M3, as reported in Table 1 (1. Neochlorogenic acid, 2. Catechin, 3. Chlorogenic acid, 4. Quercetin-3-*O*-glucoside, 5. Kaempferol-3-*O*-glucoside). Figure S3: HPLC-DAD profile of President sample—development stage P3, as reported in Table 1 (1. Neochlorogenic acid, 2. Catechin, 3. Chlorogenic acid, 4. Quercetin-3-*O*-glucoside). Figure S4: HPLC-DAD profile of Shiro sample—development stage S3, as reported in Table 1 (1. Neochlorogenic acid, 2. Catechin, 3. Chlorogenic acid, 4. Quercetin-3-*O*-glucoside, 5. Kaempferol-3-*O*-glucoside, 6. Kaempferol). Table S1: HPLC-DAD analysis—Regression equation, R2, RSD Intradie and Interdie, LOD and LOQ of standard compounds.
