# Supplementary material for: Changes in Absolute Contents of Compounds Affecting the Taste and Nutritional Properties of the Flesh of Three Plum Species Throughout Development
Source: Foods. 2019 Oct 12;8(10):486. doi: 10.3390/foods8100486 (PMC6835993; doi:10.3390/foods8100486)
Supplement: Supplementary file 1 [file foods-08-00486-s001.zip › Figure S1.docx]

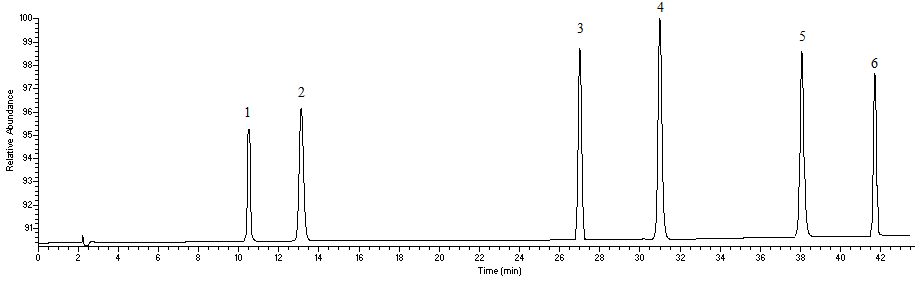


Figure S1. HPLC-DAD profile of authentic standard mixture (1. Catechin, 2. Chlorogenic acid, 3. Quercetin-3-O-glucoside, 4. Kaempferol-3-O-glucoside, 5. Quercetin, 6. Kaempferol).
