# Supplementary material for: Changes in Absolute Contents of Compounds Affecting the Taste and Nutritional Properties of the Flesh of Three Plum Species Throughout Development
Source: Foods. 2019 Oct 12;8(10):486. doi: 10.3390/foods8100486 (PMC6835993; doi:10.3390/foods8100486)
Supplement: Supplementary file 1 [file foods-08-00486-s001.zip › Figure S4.docx]

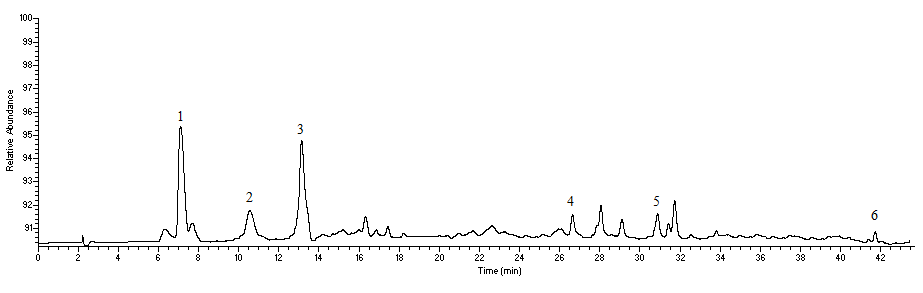


Figure S4. HPLC-DAD profile of Shiro sample - development stage S3, as reported in Table 1 (1. Neochlorogenic acid; 2. Catechin, 3. Chlorogenic acid, 4. Quercetin-3-O-glucoside, 5. Kaempferol-3-O-glucoside, 6. Kaempferol)
