# Supplementary material for: Changes in Absolute Contents of Compounds Affecting the Taste and Nutritional Properties of the Flesh of Three Plum Species Throughout Development
Source: Foods. 2019 Oct 12;8(10):486. doi: 10.3390/foods8100486 (PMC6835993; doi:10.3390/foods8100486)
Supplement: Supplementary file 1 [file foods-08-00486-s001.zip › Table S1.docx]

**Table S1**. HPLC-DAD analysis - Regression equation, R^2^, RSD Intradie and Interdie, LOD and LOQ of standard compounds

| **Standard** | **Regression**  **Equation** | | **R^2^** | **RSD***  **Intra-day** | **RSD***  **Inter-day** | **LOD** | **LOQ** |
| --- | --- | --- | --- | --- | --- | --- | --- |
|  | *Slope* | *Intercept* |  |  |  |  |  |
|  |  |  |  | % | % | μg/mL | μg/mL |
| **Catechin** | 1.84E+06 | 561405.27 | 0.9993 | 1.22 | 4.93 | 0.23 | 0.71 |
| **Chlorogenic acid** | 3.38E+06 | -5.20E+05 | 0.9996 | 0.98 | 3.71 | 0.68 | 2.17 |
| **Quercetin-3-*O*-Glucoside** | 5192200 | -162764.75 | 0.9992 | 0.48 | 2.94 | 0.53 | 1.60 |
| **Kaempferol-3-*O*-Glucoside** | 6.06E+06 | 7.29E+06 | 0.9998 | 1.13 | 3.57 | 0.71 | 2.10 |
| **Quercetin** | 9066630 | -3026230 | 0.9972 | 1.38 | 3.47 | 0.54 | 1.63 |
| **Kaempferol** | 8613450 | -2725820 | 0.9964 | 1.81 | 4.06 | 0.61 | 1.86 |

*RSD, relative standard deviation (n=4)
